# High-temperature etching of SiC in SF_6_/O_2_ inductively coupled plasma

**DOI:** 10.1038/s41598-020-77083-1

**Published:** 2020-11-17

**Authors:** Artem A. Osipov, Gleb A. Iankevich, Anastasia B. Speshilova, Armenak A. Osipov, Ekaterina V. Endiiarova, Vladimir I. Berezenko, Irina A. Tyurikova, Kirill S. Tyurikov, Sergey E. Alexandrov

**Affiliations:** 1 Academic University, Russian Academy of Sciences, ul. Khlopina 8/3, St. Petersburg, 194021 Russian Federation; 2 Peter the Great St. Petersburg Polytechnic University, St. Petersburg, 195251 Russian Federation; 3 Institute of Nanotechnology, Karlsruhe Institute of Technology, Hermann‑von‑Helmholtz‑Platz 1, 76344 Eggenstein‑Leopoldshafen, Germany; 4 Institute of Mineralogy, Southern-Urals Federal Research Center of Mineralogy and Geoecology, Ural Branch of RAS, Miass, 456317 Chelyabinsk Region Russian Federation; 5 SVCS Ltd., Zelenograd, Moscow, 124498 Russian Federation

**Keywords:** Materials for devices, Materials science, Techniques and instrumentation, Surface patterning

## Abstract

In this work, we demonstrate an effective way of deep (30 µm depth), highly oriented (90° sidewall angle) structures formation with sub-nanometer surface roughness (*R*_*ms*_ = 0.7 nm) in silicon carbide (SiC). These structures were obtained by dry etching in SF_6_/O_2_ inductively coupled plasma (ICP) at increased substrate holder temperatures. It was shown that change in the temperature of the substrate holder in the range from 100 to 300 °C leads to a sharp decrease in the root mean square roughness from 153 to 0.7 nm. Along with this, it has been established that the etching rate of SiC also depends on the temperature of the substrate holder and reaches its maximum (1.28 µm/min) at temperatures close to 150 °C. Further temperature increase to 300 °C does not lead to the etching rate rising. The comparison of the results of the thermally stimulated process and the etching with a water-cooled substrate holder (15 °C) is carried out. Plasma optical emission spectroscopy was carried out at different temperatures of the substrate holder.

## Introduction

Silicon carbide (SiC) is a wide-bandgap semiconductor material (the bandgap width is lying in between 2.36 to 3.3 eV depending on crystal structure) which has found wide application in electronic industry. Wide bandgap allows SiC based devices to be used in a very wide temperature range (up to 1000 °C). Thermal conductivity of SiC at normal conditions is close to copper which opens the opportunity to use SiC in the devices with high current densities. High thermal, radiation and chemical stability is due to the high energy of the bond between Si and C which ensures the stability of SiC-based devices under extreme operating conditions^1–5^. Thus, due to these properties, silicon carbide is a promising material for power electronics devices^6^ design as well as various microelectromechanical systems (MEMS) widely used in the automotive industry (hybrid and electric cars), energy industry, oil and gas industry, etc^7–14^.

One of the most crucial technology operations of the creation of such devices and systems is a precise formation of a profile of a given configuration (grooves, holes, etc.) on/in the silicon carbide substrate. As of today, there is a fairly large variety of techniques for the processing of silicon carbide that allow, to some extent, to solve the following problem: wet etching; etching in solvents, stimulated by femtosecond laser; plasma etching; etching in plasma atmospheric discharge; plasma jet processing, and etc^15–25^. The choice of silicon carbide processing method is determined by the specific target, but nevertheless any of the methods must meet a number of general requirements: minimal defect formation on the surface of the etched profile, high etching rates, and high directionality of the processed window during the etching process. From the above-listed methods plasma chemical etching (PCE) is one of great interest, which has found wide application in the production of various MEMS and electronic devices for some time now. Wide application of PCE is caused by a high level of process automation and ability to control a large number of process parameters, which allows to optimize the etching process for a specific task and, therefore, provides an opportunity to form various structures of a specific profile^17^. Despite the above-mentioned strengths, there are also certain drawbacks to this method. In particular, attention is still paid to the productivity increase of this technique, as well as to the problem of forming deep defect-free surfaces of windows in SiC substrates^25–28^. In this regard, interesting results were obtained in study^26^, in which the formation mechanism of the pillars at the surface of the etched openings in SiC and methods of their elimination were studied. Furthermore, in this study, it was found that the reduction in heat transfer from the etched wafer to the substrate holder, and thus, an increase in the substrate temperature due to the heating of the wafer from the plasma, leads to a significant increase in etching rate and a reduction in the roughness of the sidewalls of the etched regions. Thus, there are reasons to suppose that precise temperature control of the substrate can play a significant role in the development of an improved PCE process of SiC, suitable for the formation of deep, defect-free, vertical (with an inclination angle close to 90) structures in the material. As far as we know, no systematic work has been done to study the effect of the substrate temperature on the SiC etching rate and surface quality. Therefore, this work was aimed at studying the potential use of substrate temperature as one of the variable PCE process parameters to develop a high rate anisotropic etching process of SiC with minimal defect generation.

### Experimental details

**Figure 1:**
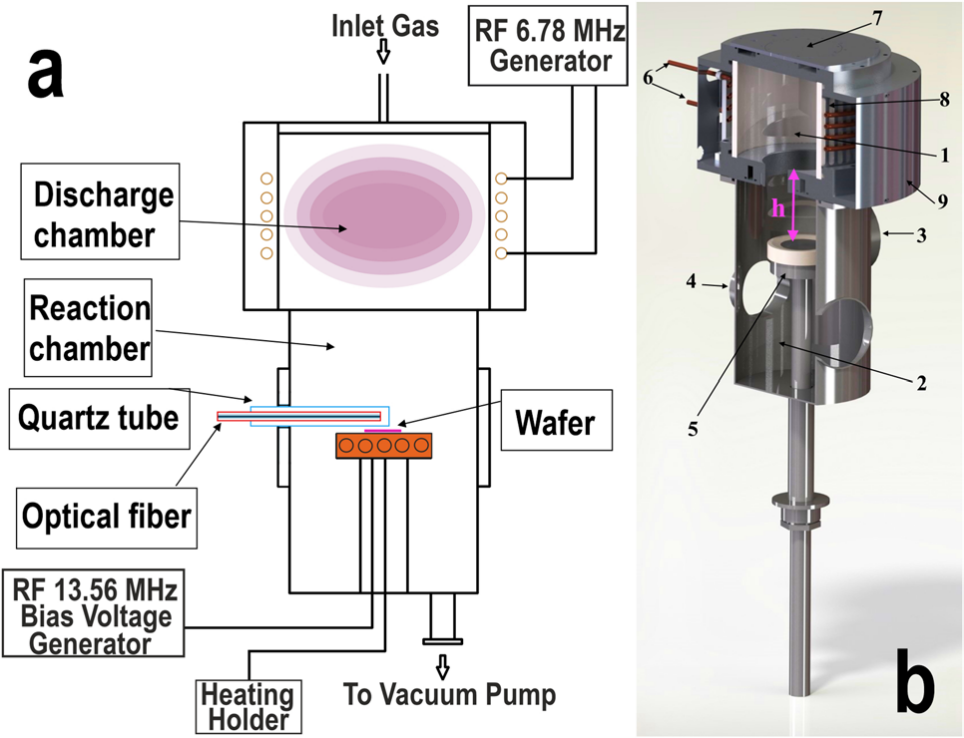
**(a)** Simplified drawing of the reactor, **(b)** 3D CAD model of PCE system reactor: (1) discharge chamber; (2) reaction chamber; (3) samples loading flange; (4) connecting flange for the pumping system; (5) substrate holder with heating unit or with water cooling; (6) water-cooled induction coil; (7) gas channel for working gas mixture; (8) Faraday screen; (9) electromagnetic screen of the induction coil.

The experiments were carried out on a custom-designed original plasma chemical etching system for various electronics materials treatment in high-density plasma (Fig. 1). For the experiments at different temperatures, the unit was equipped with a substrate holder with an internally integrated heating unit. The design of the substrate holder allowed to heat and maintain the required temperature of the substrate within the range of 20 to 400 °C^29^. To perform the etching process at 15 °C the system substrate holder was exchanged to the one equipped with a water cooling lines. The body of the substrate holder is made of corrosion-resistant stainless steel AISI 321.

As can be seen from the Fig. 1, the reactor of the system consists of discharge and reaction chambers. The reaction chamber (D_inner_ = 23 cm, H = 33.5 cm) of cylindrical shape is made of stainless steel AISI 321. Plasma in the discharge chamber (D_inner_ = 23 cm, H = 22.5 cm) was created by applying high-frequency (HF) power to the inductor of specific geometry from the HF generator (*f* = 6.78 MHz, *W*_*max*_ = 1000 W) through a resonant matching device. To form the bias potential, a 13.56 MHz HF voltage from a separate HF generator was applied to the substrate holder (electrode).

Since direct measurement and control of the processed substrate surface temperature under plasma etching conditions is itself a technically challenging task, the substrate holder surface temperature was measured in this study. The control was carried out using a type-K thermocouple located in close proximity to the lower plane of the top plate of the holder, to which a sample of silicon carbide was clamped^29^.

6H-SiC substrates with a thickness of 350 µm were used as etching samples. The standard sample size was 40 × 40 mm. In all experiments, samples were cleaned with acetone, ethyl alcohol and deionized water successively for 10 min each in an ultrasonic bath before the PCE process. In addition, after mounting the SiC substrate in the reaction chamber before the experiment, samples were treated in argon plasma for 10 min to clean up any residual contaminants from the substrate surface. The parameters of the cleaning process in argon were: applied HF power (*W* = 750 W), pressure in the reaction chamber (*P* = 0.75 Pa), Ar gas flow (*Q* = 21.75 sccm), and the distance between the bottom part of the discharge chamber and the surface of the sample (*h* = 15 cm). A low bias voltage was selected to minimize possible damage to the etching surface by energetic ions during substrate cleaning.

To improve heat transfer between the sample and the substrate holder, the SiC substrate was placed on the substrate holder through the “Ramzai” vacuum lubrication (TU 38.5901248-90). A ceramic O-ring with an outer diameter of 50 mm and an inner hole diameter of 25 mm was placed on top of the sample. Above the ceramic ring a stainless-steel hood (AISI 321) with an opening in the center (25 mm diameter) was placed to press the sample to the table. Thus, the active process region (area of etching) was a circle with a 25 mm diameter.

Sulfur hexafluoride SF_6_ (GOST TU 6-02-1249-83, purity 99.998%) was used as the etchant main gas. Etching processes were performed in a mixture of SF_6_ and O_2_ (high purity, TU 2114-001-05798345-2007). The flow rates of Sulphur Hexafluoride and oxygen were 10.15 sccm and 3 sccm, respectively. The values of HF power (*W*), bias voltage (*U*_*bias*_), pressure in the reaction chamber (*P*), the distance between the bottom part of the discharge chamber and the surface of the sample(*h*), and etching time (*t*) in all experiments were fixed and equal to 800 W, -150 V, 0.75 Pa, 15 cm and 30 min respectively. The surface temperature of the substrate holder varied from 15 (temperature of the cooling water) to 300 °C. The etching process was initiated when the substrate holder surface temperature reaches the set point.

The plasma optical emission spectrum (OES) in the chamber during the SiC etching and in the absence of SiC wafer were recorded using the OceanOpticsHR 4000 spectrometer. Spectrum were registered in the wavelength range of 200–1120 nm with a resolution of ~ 0.02 nm and an input slit width of 5 µm. The connection of the spectrometer with the system was carried out by using a fiber optic system for transmission of the plasma emission to the input slit of the spectrometer (see Fig. 1a). The spectra were processed using the SpectraGryph 1.2.14 software^30^.

After process, the etching depth and roughness of the etched surface was measured. Etching depth was evaluated from microphotographs obtained on the Carl Zeiss Supra 55VP scanning electron microscope with an accuracy of ± 2.5%, as well as direct measurement of the substrate thickness in the etched area with Micron IC 54,793 µm (measuring step is 1 µm, measurement error is 1%). In the second case, measurements were made at five different points of the substrate before and after the etching process. The etching rate (*V*_*etch*_) was calculated as the ratio of the etching depth to the process time. In addition, microphotographs were used to visually characterize the quality (roughness) of the etch surface. The root mean square roughness (*R*_*ms*_) of the surface was determined by atomic force microscopy (AFM) using the Solver-Pro NT-MDT probe microscope. Each AFM scanning area size was 10 × 10 µm. Each experiment was performed three times to prove the reproducibility of the process. The roughness was determined at five different points within each etching area. One of the measurements was made in the central point of the etching area, and the other four measurement areas were located at two mutually perpendicular diameters (two points each) at equal distances from the center of the etching area. The final roughness value was calculated by averaging the results of these five measurements. The initial *R*_*ms*_ of SiC substrates was 9.1 nm.

## Results and discussion

**Figure 2:**
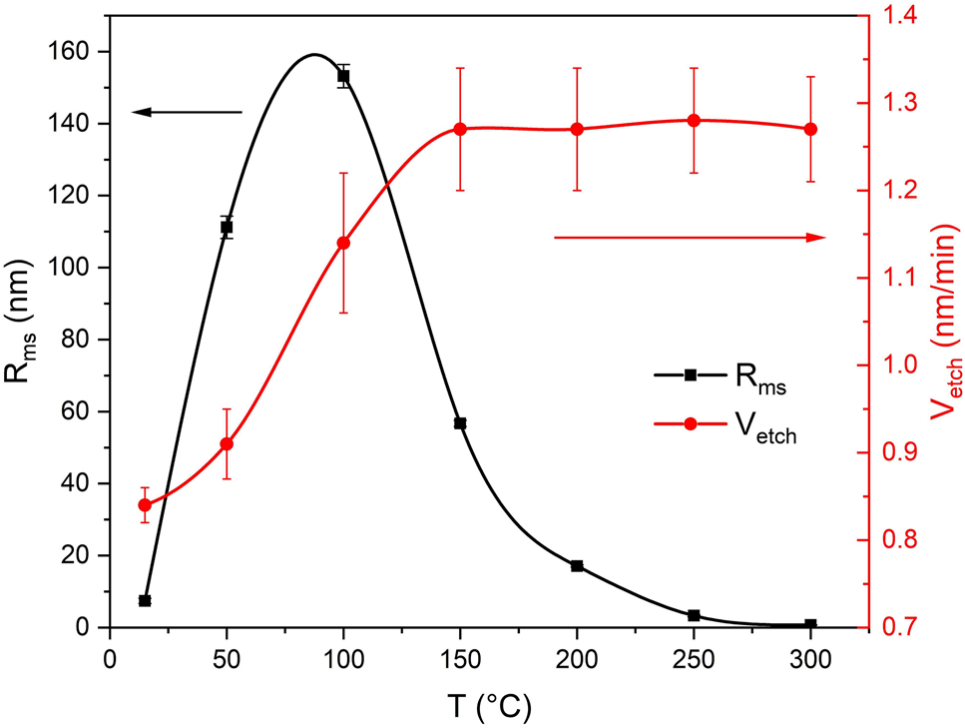
Plots of SiC etching rate (red line, circular dots) and SiC R_ms_ (black line, square dots) on substrate holder surface temperature.

The results of the study of temperature influence on SiC etching rate are presented in Fig. 2. As can be seen from the figure, the obtained dependence can be divided into two sections: I—15–150 °C and II—over 150 °C. In section I, the growth of temperature of the substrate holder leads to a noticeable and almost linear increase in etching rate with the value of increment Δ*V*_etch_/Δ*T*≈ 0.003 µm/(min·°C). Further temperature increase to 300 °C (section II) does not give any gain in the etching rate—it does not change with temperature increase.

**Figure 3:**
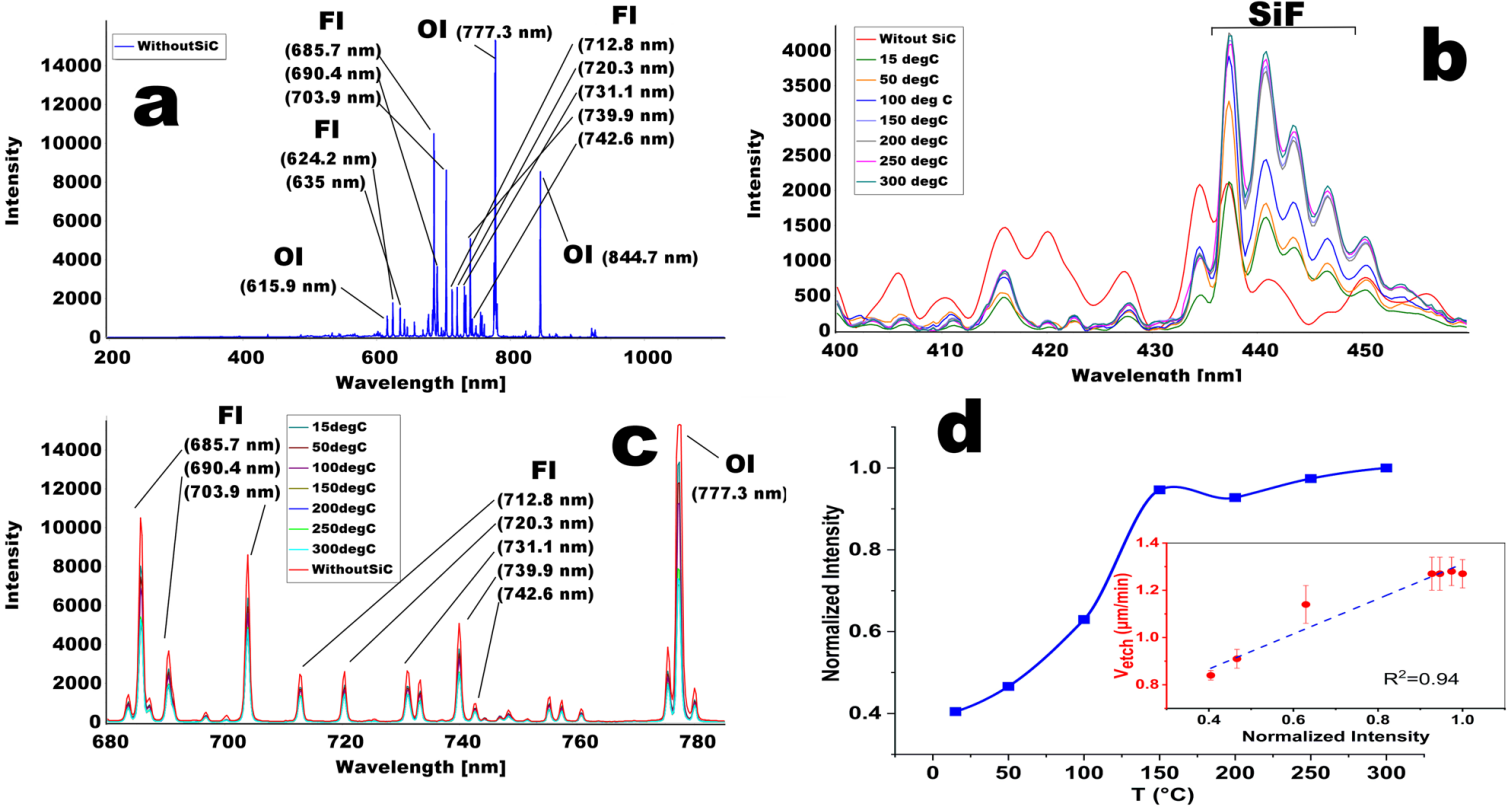
**(a)** Typical optical emission spectrum of *SF*_*6*_*(10.15 sccm)/O*_*2*_*(3.0 sccm)* plasma without SiC wafer; **(b)** 400–460 nm region of the emission spectrum of *SF*_*6*_*/O*_*2*_ plasma, registered during SiC PCE process at different temperatures; **(c)** 680–785 nm region of the emission spectrum of *SF*_*6*_*/O*_*2*_ plasma, registered during SiC PCE process at different temperatures; **(d)** normalized intensity dependence of 440. 6 nm peak on the temperature of the substrate holder during the SiC PCE process^30^.

Figure 3 presents the results of the discharge study in the reaction chamber by optical emission spectroscopy technique. Figure 3a shows the discharge emission spectrum of the SF_6_/O_2_ mixture, which was measured in the absence of SiC substrate in the chamber (no etching). The recorded spectrum contains a large number of peaks, the most intense of which are located in the wavelength range of 600–850 nm and are associated with transitions of fluorine and oxygen atoms from excited states with high energy to excited states with lower energy. The spectrum presented in Fig. 3b,c show the typical changes in the emission spectrum occurring during the SiC etching at different temperatures. From Fig. 3c, in particular, we can see that the emission intensity of excited fluorine and oxygen atoms decreases during etching of SiC. The observed decrease in the intensity is due to the active consumption of these active plasma particles during the etching of the substrate (formation of volatile SF_x_ and CO_y_). In addition, we can see that the spectrum peaks intensity of excited fluorine and oxygen atoms emission depends on the temperature of the substrate holder, namely, there is a general tendency to decrease the intensity of the spectral peaks highlighted in Fig. 3c, with a temperature increase at which the etching process was conducted. In the region of 430–450 nm (Fig. 3b), on the contrary, the substrate holder temperature rise leads to a systematic increase of emission intensity. The study of plasma emission spectra based on SiF_4_^31–33^, has shown that the group of peaks in the this range corresponds to the formation of SiF particles in the discharge. In our case, such particles are one of the possible products of the chemical reaction between fluorine radicals with SiC. In order to determine the dependence nature of the emission intensity in the range of 430–450 nm on the temperature of the substrate holder, we used the ratio *I*_T_^440.6^/*I*_300_^440.6^, where *I*_300_^440.6^—the 440.6 nm peak intensity at the temperature of the substrate holder 300 °C, and *I*_T_^440.6^—the intensity of the same peak at other temperatures. The obtained dependence is shown in Fig. 3d. It is easy to observe that the relative 440.6 nm peak intensity as a function of temperature (Fig. 3d) is qualitatively similar in nature to the etching rate dependence on the temperature of the substrate holder (Fig. 2). As in the case of *V*_*etch*_*(T)*, the *I*_*T*_^*440.6*^*/I*_*300*_^*440.6*^ ratio grows fairly rapidly when the temperature of the substrate holder changes from 15 to 150 °C and then remains relatively constant.

As known material removal during the PCE process is achieved by means of two processes: sputtering the substrate with high-energy ions (physical component of the process) and due to chemical reactions between chemically active particles (CAP) of plasma with the wafer material leading to formation of volatile compounds (chemical component of the process). As the temperature increases, the rate of chemical reactions increases, resulting in the increase of total etching rate of SiC (sections 15–150 °C in Fig. 2). This is consistent with the observed increase intensity of the SiF peaks of the plasma OES (Fig. 3d). Volatile SiF is the reaction product between the fluorine radicals and SiC. The absence of visible changes in the etching rate and in the intensity of the 440.6 nm peak in the temperature range at section II (150–300 °C) (Figs. 2 and 3d) is apparently due to the fact that after 150 °C, all CAP reaching the surface in a unit of time participate in the chemical reactions. In other words, at temperatures above 150 °C, the limiting factor for changes in the etching rate is the total CAP concentration in plasma, which does not depend on the substrate temperature but is determined by other process parameters such as chamber pressure, gas mixture composition and discharge power, which in our experiments remained constant.

During the experiments aimed at determining the dependence of etching rate on the temperature of the substrate holder, it was found that the increase in temperature above 100 °C has a positive effect on the quality of the treated surface, which is expressed in reducing the roughness of the treated surface (see Fig. 2).

**Figure 4:**
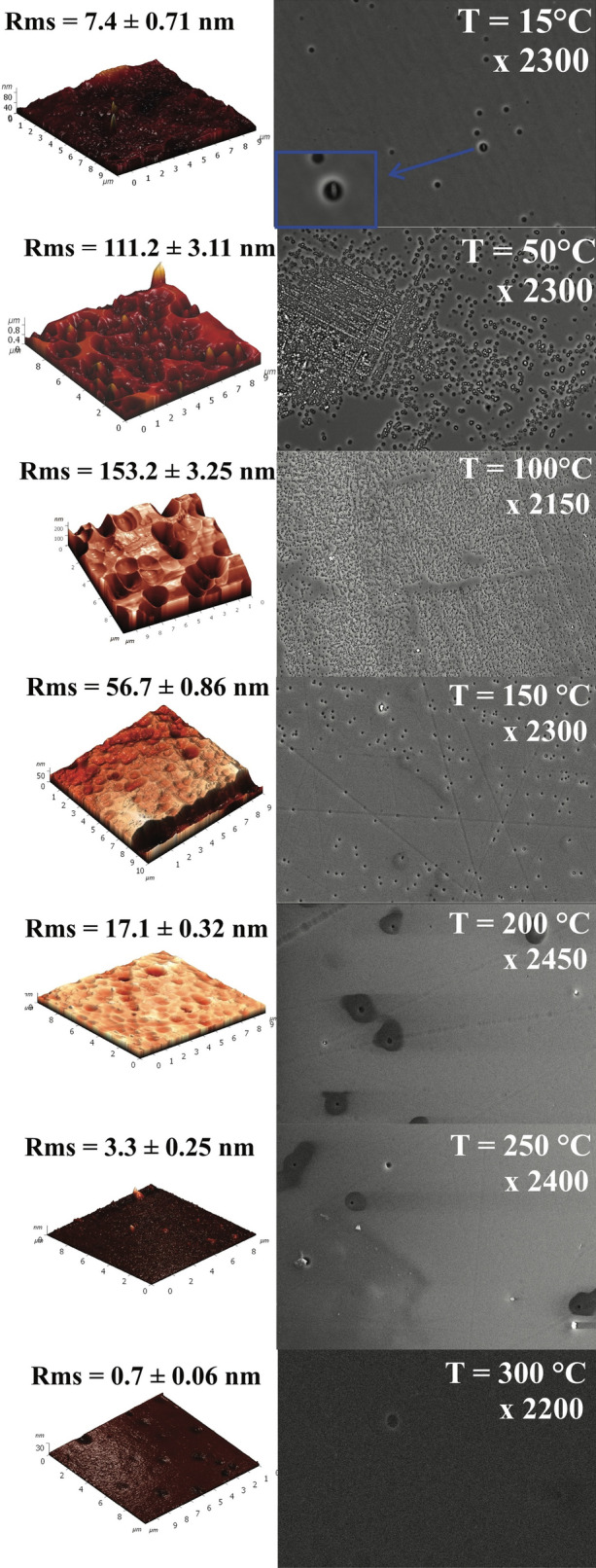
AFM results and corresponding SEM pictures made after the etching processes at different temperatures.

The results of the substrate holder temperature influence on etching surface roughness research are shown in Figs. 2 and 4. Figure 2 shows the dependence of the *R*_*ms*_ on the temperature of the substrate holder surface in the range from 15 to 300 °C, and Fig. 4 shows several microphotographs of the etching surface, taken after etching at different temperatures in the central part of the etching area. In addition, the left side of Fig. 4 shows the etching surface 3D profiles obtained by AFM investigation.

In the relatively low temperature range (T = 15–100 °C) roughness increase was observed—*R*_*ms*_ is rising from 7.4 nm to 111.2 nm at T = 50 °C, and up to 153.2 nm at T = 100 °C. Further increase in the temperature to 300 °C shows a significant reduction in sample *R*_*ms*_, i.e. with increasing temperature of the substrate itself. The detected effect can have at least three reasons: (1) an increase contribution of the chemical component on the etching process due to the temperature rise; (2) etching rate equalization of defect-free and imperfect areas of silicon carbide (imperfection of SiC substrate crystal lattice) with temperature increase; (3) the high temperature of the processed surface could eliminate the redeposition of etched products under ion bombardment. This redeposits could generate micro-masking and therefore their removing reduce the *R*_*ms*_ without increasing in the etching rate. As can be seen from Fig. 4, the most significant reduction of *R*_*ms*_ occurs when the temperature changes from 100 to 150 °C, where, according to Fig. 2, there is a significant increase in etching rate due to the increased contribution of chemical etching to the PCE process. Chemical etching, by its nature, is isotropic and, therefore, can significantly contribute to the partial or complete removal of defects in a form of various vertical structures arising on the surface during ion bombardment. The mechanism of the etching rate differentiation in defect-free and imperfect areas of the SiC substrate has been considered in the work ^26^, where it has been shown that local overheating of the etching surface in the defects location area leads to an increase in their etching rate, as a result, areas with defects are etched faster than defect-free ones. Additional heat flux from the substrate holder to the SiC wafer minimizes local overheating and thus reduces etching rate differences between different areas of the SiC wafer. Such mechanism of reduction of the etched surface *R*_*ms*_, apparently, plays a significant role at temperatures above 150 °C, as at these temperatures additional increment of etching rate due to chemical reactions of plasma’s CAP with SiC is no longer observed (see Fig. 2). Finally, evaporation of non-volatile components from the etching surface at high substrate temperatures^26^, should reduce the micro-mask effect and promote more uniform etching of SiC substrate, as well as an increase in the temperature of the substrate holder will prevent the redeposition of non-volatile components to the etching surface. As the result, the etched surface roughness should decrease.

Based on the study of the effect of temperature on the etching process behavior of silicon carbide, an additional control experiment was planned to investigate the possibility of deep vertical structures formation with low roughness by the thermally stimulated plasma etching and compare the obtained results with a similar process but performed with a water-cooled sample holder. A 350 µm thick 6H-SiC wafers with a 1 µm thick chromium (Cr) mask with windows of different geometry (stripes, circles, rectangles, etc.) and different linear sizes (100 µm to 10 mm) were used as samples. The process parameters are given in Table 1. From the table it can be seen, that values of the thermally stimulated process parameters in the control experiment corresponded to values which gave the highest etching rate with the minimum value of *R*_*ms*_. The exception is the bias voltage, the value of which was increased to improve the anisotropy of etching due to ion bombardment. When using a water-cooled substrate holder, all parameters remained the same except for the temperature of the substrate holder.

**Table 1:** Parameters of the control experiment.

*W*, W	*U*_*bias*_, V	*P*, Pa	*h*, cm	*T*, °C	*t*, min
800	− 300	0.75	15	300	25
800	− 300	0.75	15	15	25

**Figure 5:**
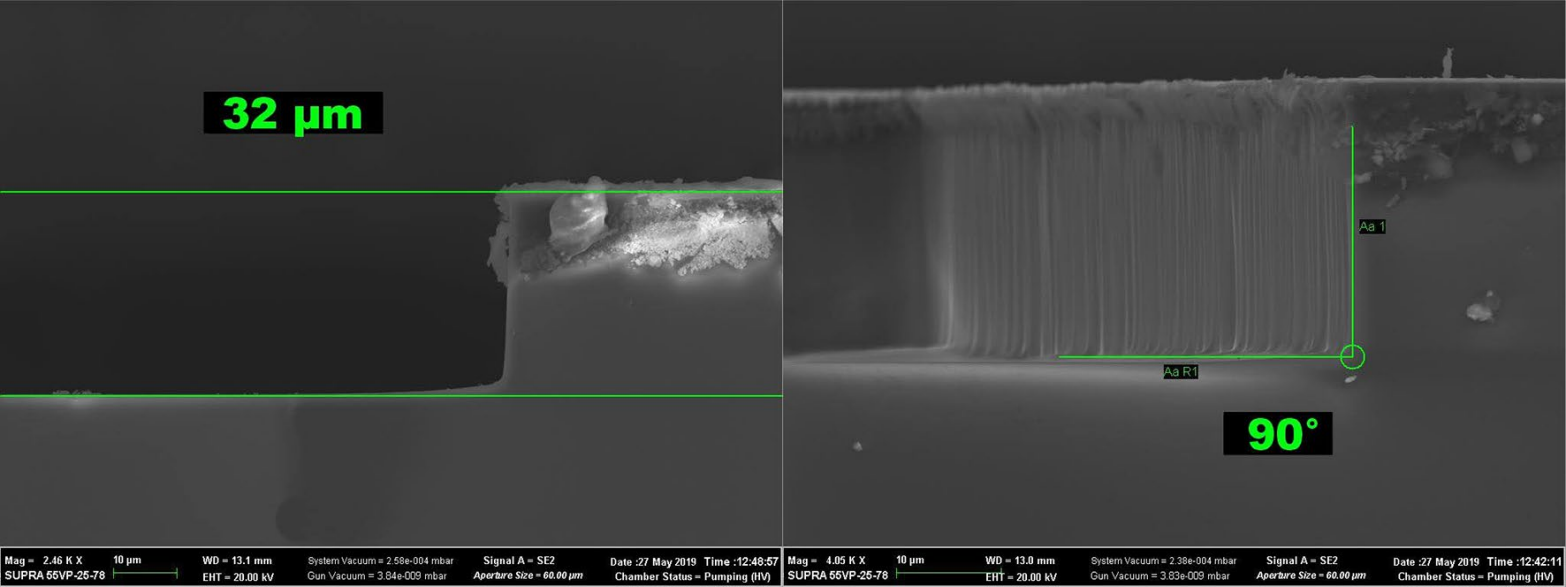
Microphotographs of the etched profile in the control experiment performed at 300 °C. (left) Cross-section view of the etched windows, (right) detailed photo of the etched window with vertical sidewall.

Microphotographs of the control experiment results are presented in Fig. 5. From the process parameters and Fig. 5, it can be seen that in this experiment the etching depth after 25 min of processing was 32 µm which corresponds to the etching rate of about 1.28 µm/min, with an angle between the etched surface and the etching profile wall of 90°. The selectivity ratio to the chromium mask was about 43.

**Figure 6:**
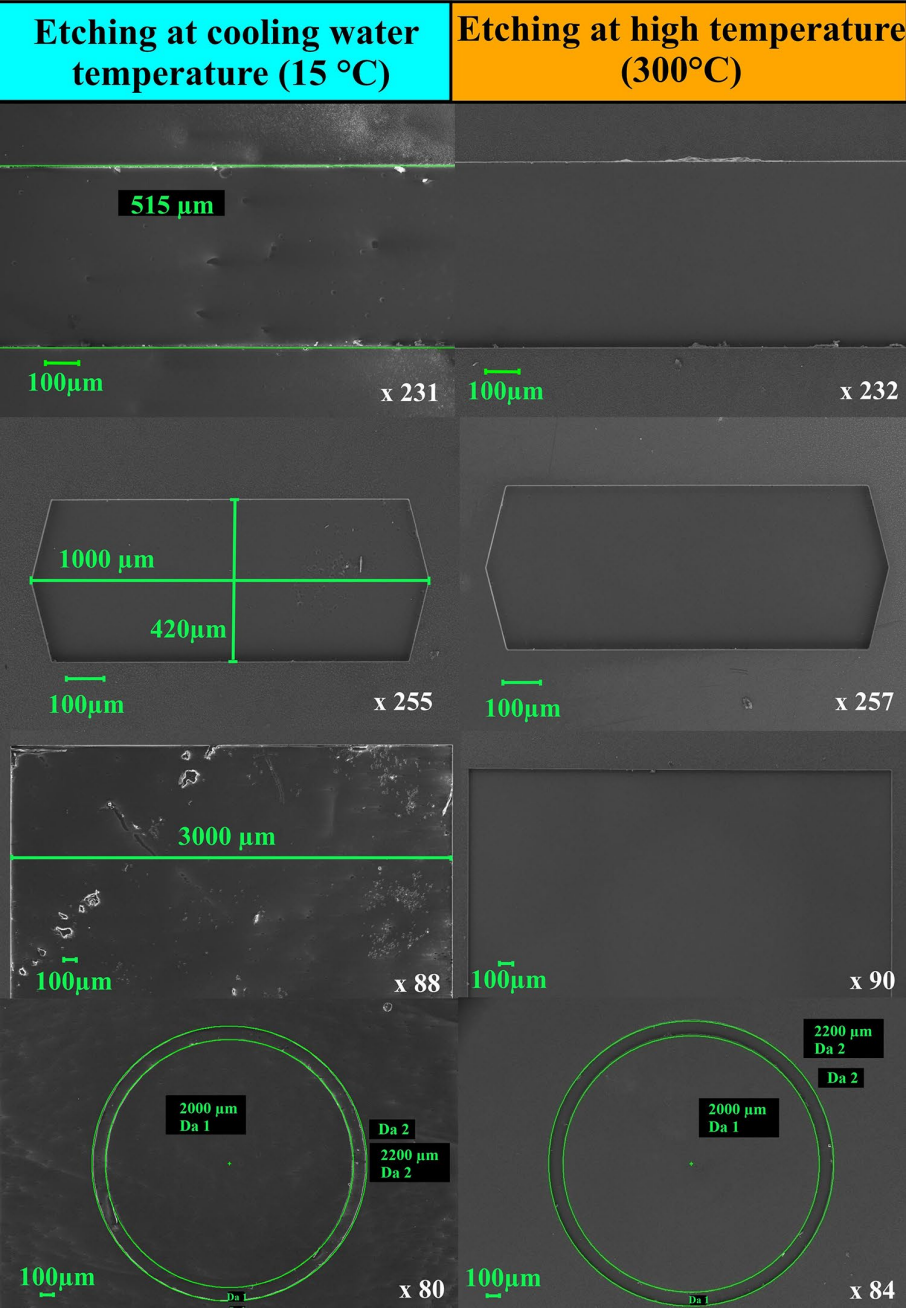
Microphotographs of the etched structures after processing at different substrate holder temperatures. Left column—etched at T = 15 °C, right column—etched T = 300 °C.

**Figure 7:**
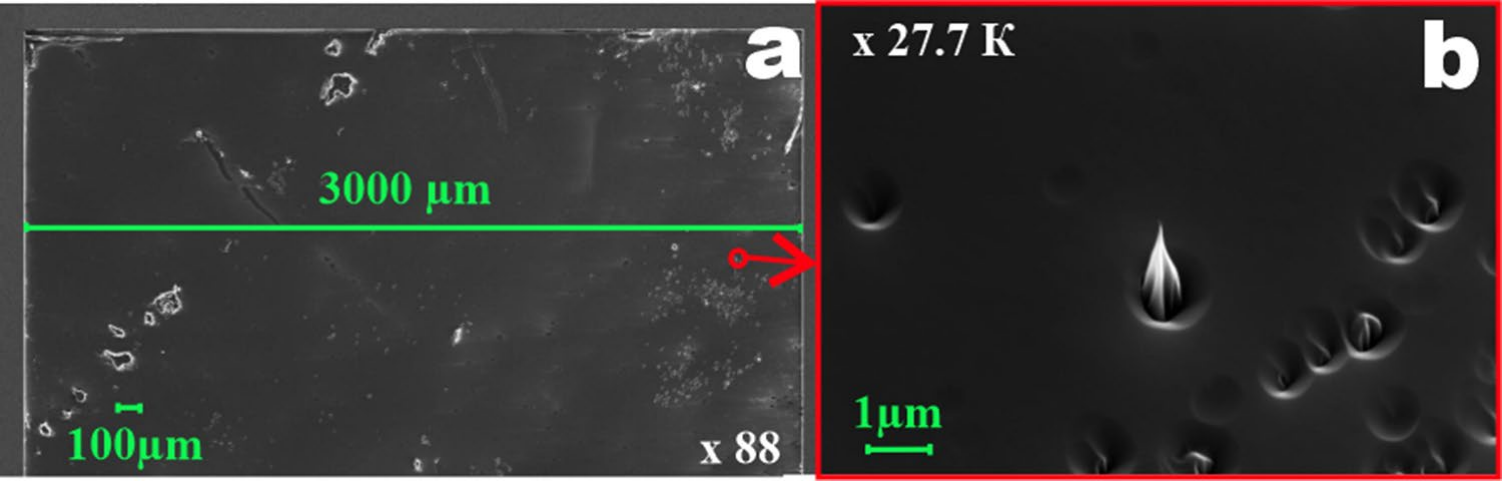
SEM microphotographs of the defects (pillars) occurring during the etching process at T = 15 °C. **(a)** Overview of the etched surface, **(b)** zoomed view of one of the appearing pillars.

In Fig. 6 top views of the obtained structures of a complicated pattern are presented. From the collected data it is visible that etching at high temperatures results in the better quality of the processed surface. The roughness of the surface etched at T = 15 °C (*R*_*ms*_ = 7.4 nm) is close to the initial SiC substrate roughness (*R*_*ms*_ = 9.1 nm) but the above mentioned, and described in ^26^, pillars appear (Fig. 7). Thus, the positive effect of the substrate temperature on the quality of etched structures can be clearly seen.

Table 2 below compares the SiC etching results obtained by various researchers, including the results of this work. Data in the table are ordered by etching rate and, as can be seen, the processes with a highest value of the etching rate were performed using plasma based on the mixture of SF_6_/O_2_. In addition, it should be noted that the first two rows in this table are processes that used additional thermal stimulation of SiC etching. In our work, as mentioned above, such stimulation was achieved by controlling the temperature of the substrate holder, and in work^26^ additional heating of the substrate was carried out due to the degradation of heat transfer conditions from the SiC substrate. The fact that we have not achieved the same high etching rate as in^26^ is obviously due to a significant difference in the value of the HF power applied to the discharge (2000 W in^26^ and 800 W in this study), and in the flow rate of the main etching gas (Q_SF6_ = 200 sccm in^26^ vs. 10.15 sccm in this study). Both these parameters to a great extent determine the CAP concentration in the plasma, and as it was considered above, the CAP concentration in the plasma is the limitation factor for etching rate in our experiments at high (over 150 °C) temperatures.

**Table 2:** The comparison of SiC etching results by different researchers.

Process characteristics		Process parameters				References
*V*_*etch*_ (µm/min)	Angle	*W* (W)	*P* (Pa)	*U*_*bias*_	Gas mixture	
2	~ 90°	2000	5	200 W	SF_6_/O_2_	^26^
1.28	~ 90°	800	0.75	− 300 V	SF_6_/O_2_	This work
> 1	~ 98°	1000	0.67	300 W	SF_6_/O_2_/Ar	^18^
0.97	–	900	0.8	− 450 V	SF_6_/O_2_	^34^
0.94	~ 117°	1000	–	250 W	SF_6_/O_2_	^19^
0.6	~ 88°	700	0.67	− 400 V	SF_6_/O_2_	^35^
0.55	–	1500	0.8	− 300 V	SF_6_/O_2_	^25^
0.5	~ 98°	1000	0.67	120 W	SF_6_/O_2_/Ar	^36^
0.4	–	400	33	− 100 V	SF_6_/Ar	^37^
0.36	~ 85°	900	1.3	− 300 V	SF_6_	^38^
0.31	~ 90°	2000	2.7	200 W	SF_6_/O_2_	^17^
0.23	~ 100°	1000	0.5	− 160 V	Cl_2_/Ar	^39^
0.22	–	800	0.67	75 W	SF_6_/Ar	^40^
0.22	–	300	1.1	250 W	Cl_2_/Ar/BCl_3_	^41^

The *U*_*bias*_ values are given in those units in which they were presented in the original work.

## Conclusions

The influence of substrate holder temperature on the etching rate and the surface roughness of plasma-chemical etching of the SiC in SF_6_/O_2_ gas mixture has been studied. It is demonstrated that as the etching temperature increases, the etching rate increases monotonically up to the moment when other factors (CAP concentration in our case) become a limiting factor with respect to the etching rate. With a help of the optical emission spectroscopy technique it was shown that with the substrate holder temperature rise the SiC etching rate increase proportionally to the change of the SiF (440.6 nm) peak. Etched surface roughness decreases with the temperature rise above 100 °C, which may be due to the increased contribution of the isotropic chemical etching by CAP of plasma and equalization of etching rates of defect-free and imperfect SiC regions. Also, decrease in non-volatile compounds passivation of the surface and the micro masking effect with temperature rise. Thus, the choice of the optimum substrate holder temperature mode allows to significantly increase the etching rate of the process and reduce surface roughness of the final structure. In particular, we achieved etching of the vertical structure of a complicated pattern with etching rate of about 1.28 µm/min at the substrate holder temperature of 300 °C using relatively low HF power of 800 W. The root mean square roughness of the processed substrate surface was about 0.7 ± 0.06 nm. It has also been demonstrated that thermally stimulated etching does not result in the formation of pillars, which is typical for etching in fluorinated based plasmas at the low substrate holder temperatures. This result demonstrates a high potential of using thermally stimulated etching processes to solve the problem of "dry" processing of SiC in plasma.
